# The Climatology of England

**Published:** 1890-10-25

**Authors:** 


### CLIMATOLOGY.

### CLIMATOLOGY OF ENGLAND.

Mr. Bertham Thornton, surgeon to the Royal Hospital for Scrofula at Margate, communicates to a contemporary, remarks on the climatology of London, as compared with a large number of so-called health resorts. He regrets, however, that there are not sufficiently accurate meteorological datæ. Some of those published are the work of amateurs, many are intermittent, recording instruments are not always similar, the position of instruments varies fatally, and there is reason to believe some evidence is untrustworthy. It might also be added, that, as with other statistics, so meteorological statistics may be manipulated to serve a purpose. Dr. Thornton has rejected all observations not made in accordance with the regulations of the Royal Meteorological Society. The climate of a locality cannot be estimated from mere thermometrical observations. Winds, rainfall, humidity, soil, vegetation, aspect, and surrounding physical conditions must all be taken into account. Of some thirty so-called

"health resorts," the mean summer temperature, as shown by the records of a number of years, was highest (57·6°) at Guernsey, and lowest (56·1°) at Bath. The winter temperature, highest (45°), at Guernsey, and lowest (40·2°) at Bath. Figures midway represent the temperature of the Regent's Park. The mean daily range was lowest at Ilfracombe (8·4°), and highest at Cheltenham (15·4°). At the Regent's Park it was 13.8°. At Bath there was the highest degree of humidity. But all places exposed to the breezes from the Atlantic, have a humid atmosphere. The saturation of the atmosphere with aqueous vapour was lowest in the City of London, the figures being 77 for the City, 80 for the Regent's Park, and 86 for Bath. Wind records, Mr. Thornton says, are difficult to make out, and this may be readily imagined as local conditions, such as buildings, direction of streets, trees, hills, &c., all interfere. The amount of sunshine is also difficult to appreciate, for many obstructions make a difference to the actual amount of sun rays poured on any one particular spot. A perusal of Mr. Thornton's paper leaves an impression that there is no special paradise of health, and we fully endorse the author's observation that invalids in the season months are liable to fare badly as regards comfort and quiet at such haunts as Eastbourne, Ramsgate, Margate, Brighton, and Scarborough. The author also gives another caution which may be endorsed, viz., that invalids should not be lured by a bright sun into depressing biting east winds. A combination which is found more or less not only in too many English health resorts but also in the Riviera.

We mentioned above that Mr. Thornton might have added to his difficulties, that meteorological statistics can be manipulated to suit local views. To suit local purposes and views, one spot, as Mr. Thornton remarks, is depicted as a Monte Carlo, while another not far away is stigmatised as a Siberia. As a matter of fact, those who gain a living and flourish in a place are frequently unduly biassed in favour thereof. Mr. Thornton himself is scarcely quite free from this, for he says—what is perhaps perfectly true of almost any place—that invalids, even in the winter, will find warm localities at Margate. Climate affects people in many ways. Perhaps the most important manner in which it acts is by the amount of moisture in the atmosphere, for this influences the evaporation from both lungs and skin. This is of more importance to many invalids than thermometrical range. A cold dry atmosphere is often borne with comfort, when the same degree of cold, accompanied by moist air, would produce inconvenience. A similar remark applies to hot dry, and hot moist air. This is the principal reason why such places as Davos Platz, Maloya, Pontresina, &c., suit so many invalids, notwithstanding that the salubrity of such climates has been attributed to the absence of the fashionable microbe. But the question whether a particular climate will suit an invalid can scarcely be decided altogether on the characteristics of the climate. There are the individual peculiarities of the patient. Some people, whether well or ill, feel hot and cold, moisture and dryness, much more acutely than others. Such peculiar idiosyncracies should be taken into consideration. Lastly, it may be observed that a study of Mr. Thornton's interesting communication leads to the conclusion that English climate does not differ so much as might be supposed, even in localities far apart. Neither are the drawbacks of the English climate such as would justify that decided jubilation made in favour of certain other localities, where the only advantage is more sunshine. But sunshine is not everything. Warmth, dry atmosphere, sunshine, an emerald sea, and flowers in winter, as on the Riviera, are very good things. But even many spots thus favoured are liable to drawbacks, such as a hot sun and a cold shade, a great evening fall of temperature, or cold northerly winds blowing over high snow mountains. As regards this London of ours, if we could only get rid of the smoke-fog-demon, we question if there would be a better climate in the whole of the British Isles.

